# The distal tibiofibular joint effusion may be a reliable index for diagnosing the distal tibiofibular syndesmosis instability in ankle

**DOI:** 10.1007/s00256-023-04395-4

**Published:** 2023-07-19

**Authors:** Shouqi Sun, Chao Chen, Zhuoqi Sheng, Min Wei

**Affiliations:** 1 Medical School of Chinese PLA, Beijing, China; 2 Department of Orthopedics, the Fourth Medical Center, Chinese PLA General Hospital, Beijing, China; 3 Department of Orthopedics/Chinese National Clinical Research Center for Orthopedics, Sports Medicine and Rehabilitation, Chinese PLA General Hospital, Beijing, China; 4 Department of Rehabilitation Medicine, the Second Medical Center & National Clinical Research Center for Geriatric Diseases, Chinese PLA General Hospital, Beijing, China

**Keywords:** Arthroscopy, MRI, Distal tibiofibular joint effusion, Distal tibiofibular syndesmosis instability, Retrospective, Diagnostic-accuracy study

## Abstract

**Purpose:**

To analyze the accuracy of MRI in diagnosis of distal tibiofibular syndesmosis instability (DTSI) and construct new diagnostic parameters.

**Materials and methods:**

This retrospective study evaluated 212 patients with history of ankle sprains and 3 T MRI and received a final diagnosis of distal tibiofibular syndesmosis instability by ankle arthroscopic surgery from October 2017 and December 2021. We compared the accuracy of syndesmotic injury, qualitative index of distal tibiofibular joint effusion (DTJE), and quantitative index of distal tibiofibular joint effusion (DTJE) in diagnosing distal tibiofibular syndesmosis instability. The criteria for syndesmotic injury were consistent with previous literature, and DTJE was grouped according to the pre-experimental results.

**Results:**

A total of 212 patients (mean age, 35.64 ± 11.79, 74 female and 138 male) were included. Independent predictive MRI features included syndesmotic injury, qualitative index of distal tibiofibular joint effusion, and quantitative index of DTJE including the height, projected area of equal-point method, and projected area of incremental-value method. The quantitative index of DTJE showed a higher area under the receiver operating characteristic curve (0.805/0.803/0.804/0.811/0.817/0.805 > 0.8, *P* < 0.05; in comparison with all other method). The height measurement method was simpler and easier to operate, that could be gotten only by measuring the DTJE distance of a MRI independent layer, and the cut-off value of the effusion height was 8.00 mm and the Youden index (0.56) was the best.

**Conclusions:**

Our research translated a complicated string of MRI multi-dimensional spatial measurements into a simple measuring process, and established the significance of quantifying DTJE in the diagnosis of DTSI. We found that the 8-mm height of DTJE was a more specific indicator for DTSI and could serve as a novel MRI diagnostic cutoff in clinical practice.

## Introduction

Distal tibiofibular syndesmosis injury, or high ankle sprain, is a frequent athletic trauma [1]. A previous study suggested that 20.3% of ankle sprains had concomitant distal tibiofibular syndesmosis injury [2]. Distal tibiofibular syndesmosis involves the anterior inferior tibiofibular ligament (AITFL), posterior inferior tibiofibular ligament (PITFL), tibiofibular interosseous ligament (TFIL), and transverse tibiofibular ligament (TTFL) and plays a crucial part in maintaining the stability of the ankle joint [3].

The distal tibiofibular syndesmosis injury tends to be disregarded due to inadequate knowledge, lack of awareness, and even misdiagnosis. Against the West Point Ankle Grading System, the distal tibiofibular syndesmosis injury falls into three categories [4]. Type I injury, or strain type, in which syndesmosis is stable, is treated conservatively, while type III injury, or separation type, where the syndesmosis is fully disrupted or fractured, requires surgical stabilization. The intermediate type, i.e., type II injury (instability type), in which syndesmosis injury is atypical, takes more time to decide if it is indicated for surgical management since joint instability has been so vaguely defined. In view of this, we dubbed type II injury as distal tibiofibular syndesmosis instability (DTSI) [5].

MRI is the most reliable method used to prevent missed diagnosis of type I and II distal tibiofibular syndesmosis injuries compared with X-ray [6–8]. The positive findings of distal tibiofibular syndesmosis injury by MRI, as specified in previously reported criteria, include (1) absence of syndesmotic ligament; (2) abnormal course or wavy, irregular thickening of the distal tibiofibular ligament; (3) increased signals of the distal tibiofibular ligament on T1- and T2-weighted images on MRI; and (4) high-intensity signals inside the distal tibiofibular joint seen on coronal MRI [7, 9]. DTSI usually shows incomplete ligament damage. However, it is still uncertain whether these standards can diagnose DTSI well.

In clinic, we found that the distal tibiofibular joint effusion (DTJE) and the amount of fluid from the mortise into the syndesmosis could well serve as specific indicators for more accurate diagnosis of DTSI. The purpose of this study was to retrospectively determine the value of DTJE, as a diagnostic indicator, in MRI, against syndesmosis injury, with the arthroscopic finding as the reference standard.

## Materials and methods

This retrospective single-center study was approved by the Ethics Committee (2021–637). The requirement to obtain informed consent was waived because of the retrospective nature of the study.

### Study patients

**Fig. 1:**
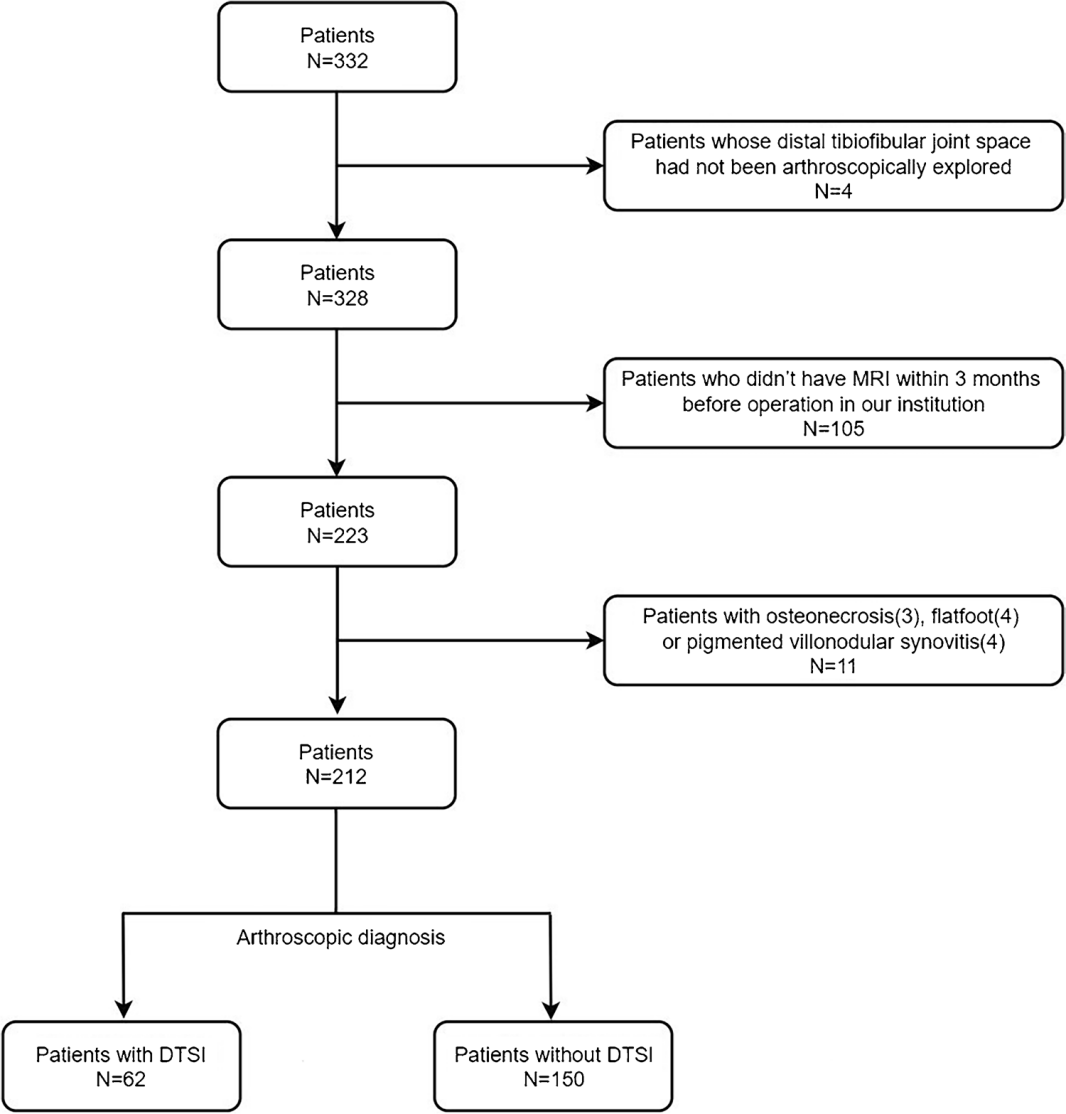
Flow chart of patient recruitment

We performed a retrospective review on consecutive patients with ankle sprains arthroscopically diagnosed as having DTSI who were referred to a Sports Medicine wards between October 2017 and December 2021. Inclusion criteria were (1) patients suffering from ankle sprain and (2) patients receiving ankle arthroscopic procedure and preoperative MRI within 3 months of the surgery. Exclusion criteria included (1) patients whose distal tibiofibular joint space had not been arthroscopically explored, (2) those who did not have MRI within 3 months before operation in our institution, and (3) those with other ankle abnormalities, including osteonecrosis, flatfoot, and pigmented villonodular synovitis (Fig. 1).

#### MRI

All MRI examinations were performed on a 3-T imager (SIEMENS; Magnetom Skyra) and our routine MRI protocol [T1-weighted (TR/TE, 450/13 ms; matrix, 512*512) and T2-weighted (TR/TE, 3700/68 ms; matrix, 512*512)] based on dual spin-echo sequences in which the slice thickness was 3 mm without any intersection gap. Other important indicators of MRI examination included that the orientation was from head to toe, each sequence needed 2 min, the coils were special coils for an ankle joint, and there are eight channels. All MRI results were interpreted by means of a Medcare picture archiving and communication system.

MRI images were evaluated, by two radiologists, who, respectively, had 6-year and 10-year experiences with musculoskeletal MRI, and were not informed of the patients’ clinical history and arthroscopic results.

### Syndesmotic injury

**Fig. 2:**
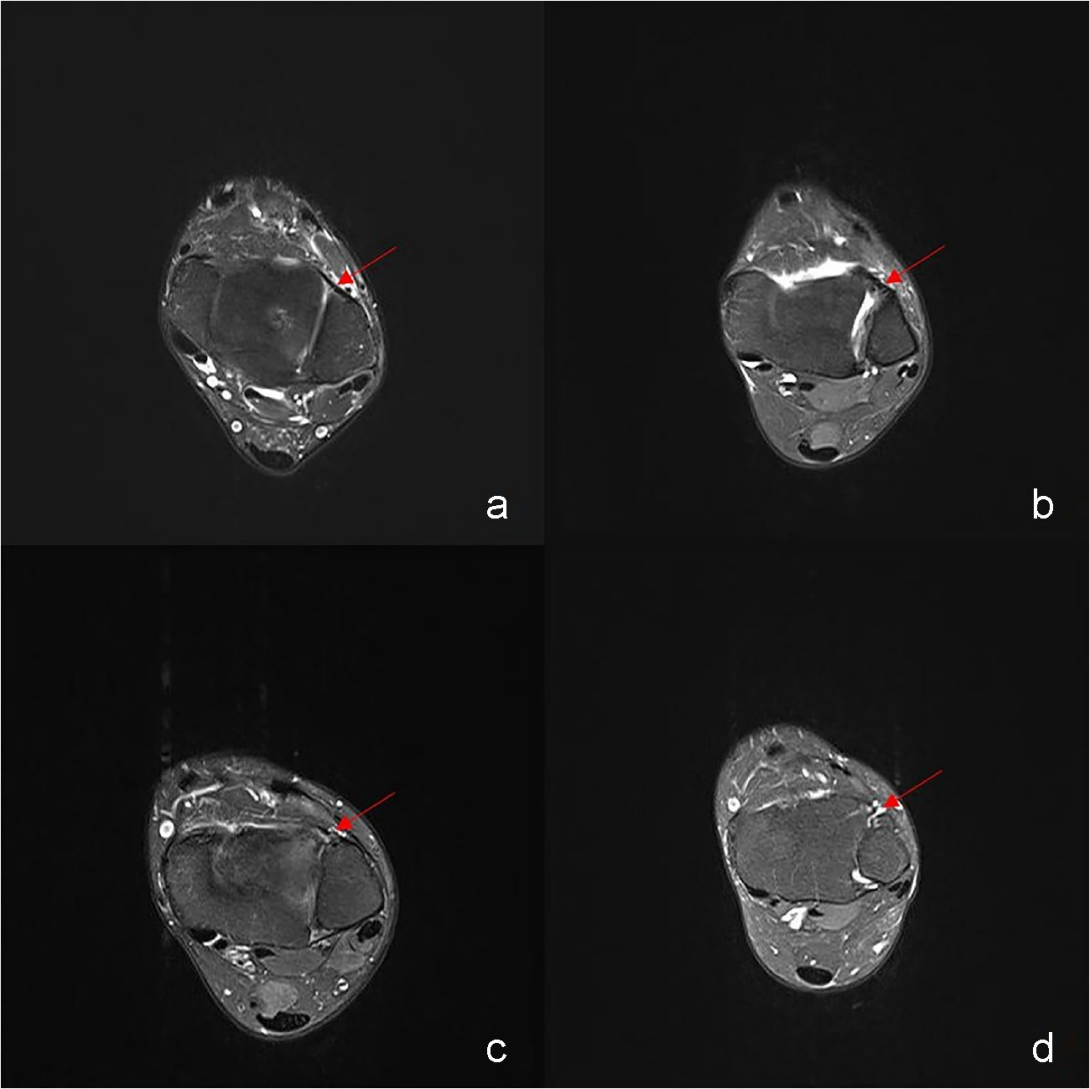
**a**–**d** Imaging findings of adjusted and non adjusted AITFLs on a route MRI protocol (T2 weighted images obstructed approximately at level of tidal flat on horizontal level/plane). When AITFL did not exhibit discontinuity or thickening, it was considered not adjusted (arrow in **a**, a score of 0). AITFL was seen as being adjusted when it was thick (arrow in **b**, a score of 1), with a wavy contour or discontinuity (arrow in **c**, a score of 2), or absent (arrow in **d**, a score of 3). AITFL, anterior inferior tibiofibular ligament

MR images were evaluated for the presence of syndesmotic injury and osseous or chondral ankle injuries [10, 11]. For injury of the AITFL and PITFL, each observer rated the syndesmosis injury on a four-point scale that indicated the probability of ligament injury. With the scale, a score of 0 meant that the syndesmosis was definitively intact (Fig. 2a); a score of 1 was indicative of probable injury of the syndesmosis, with hyperintense signals found on the T2-weighted images, suggesting traumatic edema or the chronic thickening (Fig. 2b); a score of 2 showed that the syndesmosis might sustain partial tear, as indicated by a wavy or curved ligament (Fig. 2c); a score of 3 signified that the syndesmosis was definitively injured, with complete discontinuity (Fig. 2d). Based on previous literature and clinical experience, we took scores of 2 and 3 as positive MR imaging results for DTSI [17].

### Distal tibiofibular joint effusion (DTJE)

#### Qualitative index of DTJE

**Fig. 3:**
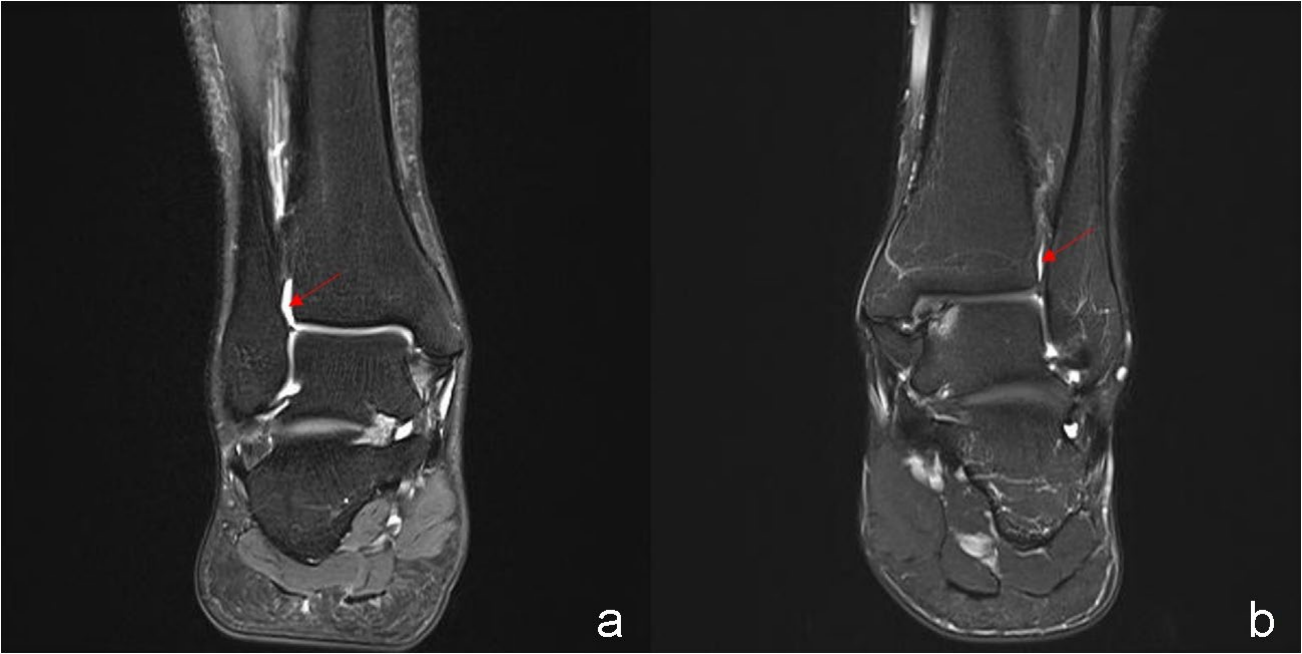
DTJE in coronal position of ankle joint. Communication group (red arrow in **a**) and non-communication group (red arrow in **b**) are in terms of whether the distal tibiofibular space was communicated with the ankle cavity. DTJE, distal tibiofibular joint effusion

The distal tibiofibular joint was observed for the effusion on the coronal plane of the ankle joint (Fig. 3a and b). We categorized joint effusion into two types, i.e., a communication group (Fig. 3a) and a non-communication group (Fig. 3b), in terms of whether the distal tibiofibular space was communicated with the ankle cavity.

### Quantitative index of DTJE

With the communication type, the penetration of the distal tibiofibular joint and ankle joint was found in at least one coronal MRI image, while with non-communication type, no evidence of communication between the distal tibiofibular joint and the ankle joint was found on any coronal MRI images.

#### Height of DTJE

**Fig. 4:**
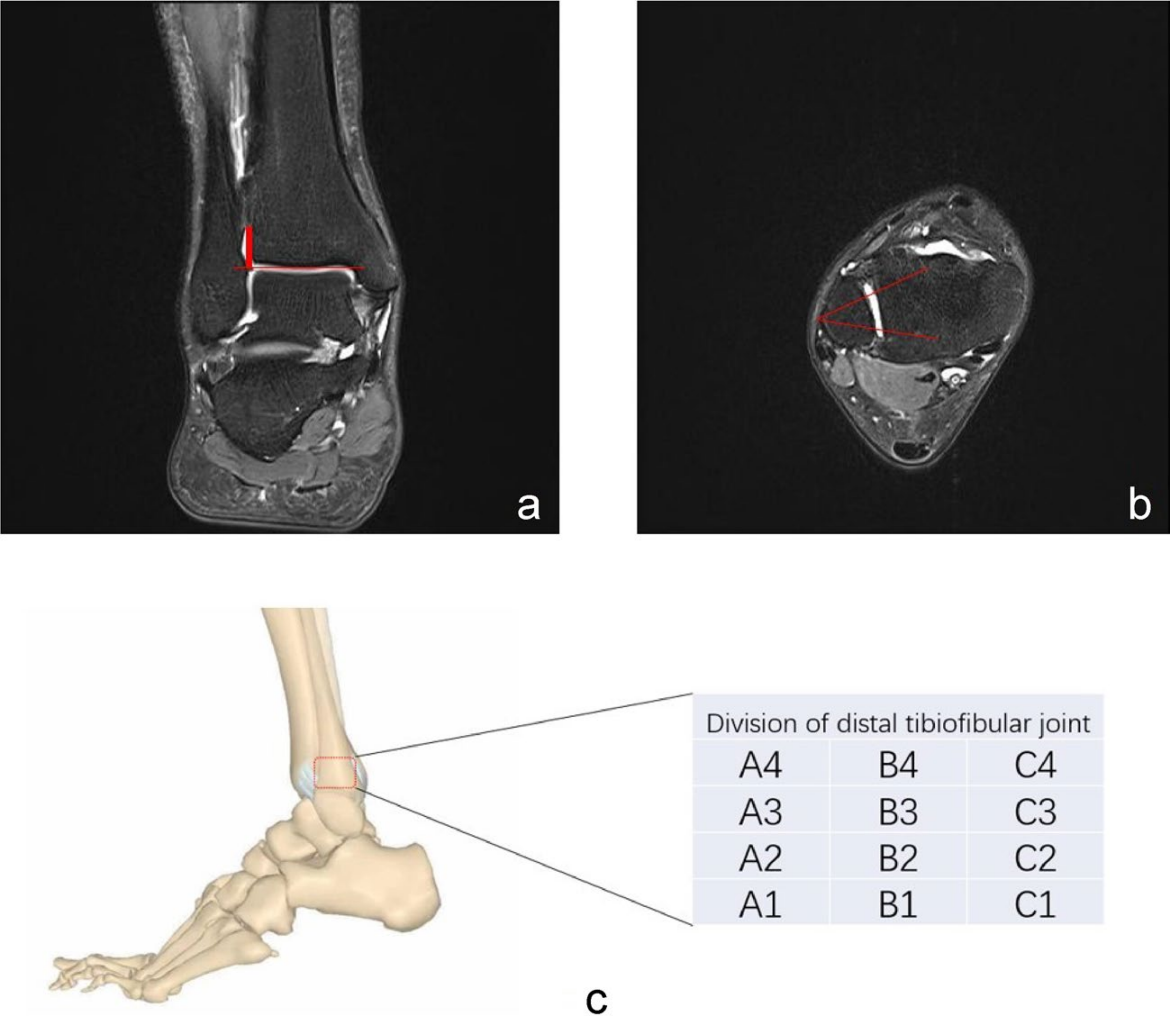
Measurement of the height of DTJE (**a**) and projected area of DTJE (**b** and **c**). DTJE, distal tibiofibular joint effusion

On the coronal plane, a line was made along the tibial plafond surface, and a line perpendicular to the line was drawn, going through the highest point of joint effusion (Fig. 4a). Then the height was measured and the maximal value was taken. The height was taken as 0 when no effusion existed in the distal tibiofibular joint.

#### Projected area of DTJE

(1) On the coronal plane, a line perpendicular to the baseline of the articular surface of tibia was drawn via the highest point of DTJE. From the intersection point up, four segments were taken along the vertical line (each segment being 4 mm long), serving as *Y*-axis (Fig. 4a). (2) Horizontally, the tibiofibular joint was equally divided into three segments, serving as X-axis (Fig. 4b). (3) On the saggital plane, the tibiofibular joint could be projected onto an imaginary plane consisting of 12 areas (boxes).

We used two methods to calculate the projected area of DTJE, i.e., the “equal-point” method and the “incremental-value” method. With equal-value method, a point was awarded if a box contained fluid. For the incremental value method, the point value increased progressively up the *Y*-axis, for instance, on the level 1 (A1, B1, C1), a point was awarded if any box had fluid; 2 points were awarded if any box at the level 2 (A2, B2, C2) had fluid and so forth, with the maximal score (points) being 12 (3 × 4).

### Reference standard

**Fig. 5:**
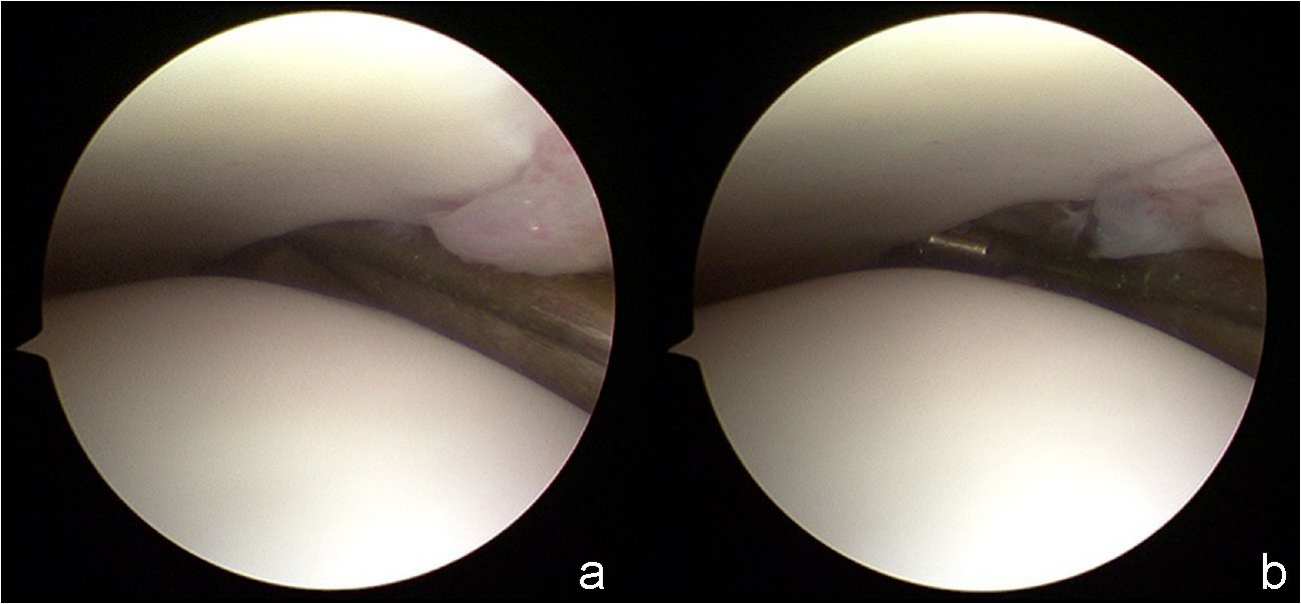
Arthroscopic examination of the tibiofibular space. Process: Push the probe tip into the distal tibiofibular joint space (**a**). The probe tip opens the distal tibiofibular joint space (**b**)

Arthroscopy is considered to be the gold-standard of reference for the evaluation of DTSI. Arthroscopic exploration: Briefly, the probe tip was inserted into the distal tibiofibular joint space (Fig. 5a), with the hook being rotated axially. If the probe tip could open the distal tibiofibular joint gap (Fig. 5b).

When DTSI was positive on arthroscopy, the probe tip could open the distal tibiofibular joint gap. That meant the distal tibiofibular joint space was greater than 1 mm and the ankle joint was instable [12].

The operation was done by a sports medicine doctor with 30 years of clinical experience.

### Statistical analysis

The statistical analysis was performed by using the Statistical Package for Social Sciences software (SPSS, version 26.0). Measurement data included syndesmosis injury score, height, and projected area score of DTJE. Measurement data were presented as mean ± standard deviation (SD) and were rounded to two decimal places. The independent sample *t* test was used for measurement data and chi-square test was employed for enumeration data. Alpha (*α*) value was set at 0.05.

Kappa tests (*κ* tests) were performed to assess interobserver variability between both the readers in judging syndesmosis injury and qualitative index of DTJE. The degrees of agreement were categorized as follows: a *κ* value of less than 0.00 indicated poor agreement; a *κ* value of 0.00–0.20, slight agreement; a *κ* value of 0.21–0.40, fair agreement; a *κ* value of 0.41–0.60, moderate agreement; a *κ* value of 0.61–0.80, substantial agreement; and a *κ* value of 0.81–1.00, almost perfect agreement [13]. Inter-observer variability for quantitative index of DTJE was assessed using Bland–Altman graphs, and bias and limits of agreements for each measured value were given in millimeters [14].

A series of 2 × 2 contingency tables were generated, using the arthroscopic diagnosis (positive or negative for DTSI) as the reference standard. Sensitivity, specificity, Youden index, and their 95% CIs were calculated for distal tibiofibular joint effusion (DTJE) as well as for the positive clinical diagnosis [15].

A multi variable analysis model with fixed and independent factors was used to test the diagnostic accuracy of AITFL, PITFL, and DTJE for DTSI.

The Bland–Altman graph was used to test the observation uniformity of height of DTJE and projected area of DTJE among different observers.

ROC analysis was utilized to assess syndesmotic injury, i.e., the height and the projected area of DTJE. The area under the curve (AUC) and the optimal cut-off value for each parameter were determined by using Youden index. Discriminatory power of the ROC curves was interpreted as excellent (if AUC = 0.9–1); good (if AUC = 0.8–0.89); fair (if AUC = 0.7–0.79); poor (if AUC = 0.6–0.69); and null (having no discriminatory power) (if AUC = 0.5–0.59). Alpha (*α*) value was set at 0.05 [16].

## Results

### Subject characteristics of patients

Arthroscopic results confirmed that 62 of the 212 ankles had distal tibiofibular syndesmosis instability. In terms of the arthroscopic results, the patients were divided into two groups, i.e., group with DTSI and group without DTSI. Patients’ data, including age, gender, side, height, weight, and BMI, are given in Table 1. For the patients, additional arthroscopic diagnoses were as follows: synovitis, chronic lateral ankle syndesmosis injury, and osteochondral lesion of the talus or tibia (Table 1).

**Table 1:** General features of the patients

ITEM	Total (*N* = 212)	With DTSI (*N* = 62)	Without DTSI (*N* = 150)	*P*
Age(year)	35.64 ± 11.79	32.98 ± 10.23^*^	36.73 ± 12.24^*^	0.035
Gender(male:female)	138 (65.09%):74 (34.91%)	29 (46.77%):33 (53.23%)^*^	109 (72.67%):41 (27.33%)^*^	0.000
Side(left:right)	100 (47.17%):112 (52.83%)	28 (45.16%):34 (54.84%)	72 (48%):78 (52%)	0.706
Height (m)	1.71 ± 0.08	1.70 ± 0.08	1.72 ± 0.08	0.080
Weight (kg)	74.50 ± 13.57	70.81 ± 14.18^*^	76.01 ± 13.06^*^	0.011
BMI (kg/m^2^)	25.26 ± 3.48	24.45 ± 3.73^*^	25.60 ± 3.33^*^	0.028
Complications				
Synovitis	23	12	11	
Osteochondral lesion of the talus or tibia	41	4	37	
Chronic lateral ankle syndesmosis injury	68	29	39	
Osteochondral lesion of the talus or tibia + Chronic lateral ankle syndesmosis injury	80	17	63	

*DTSI*, distal tibiofibular syndesmosis instability; *BMI*, body mass index. *P < 0.05

### Diagnostic accuracy of syndesmotic injury for DTSI

The *κ* value representing the degree of interobserver agreement for the two readers with the AITFL injury was 0.837 (almost perfect agreement) and *κ* value representing the degree of interobserver agreement for the two readers with the PITFL was 0.819 (almost perfect agreement). We used ROC to judge the diagnostic accuracy of syndesmotic injury for DTSI. On the basis of the results of both two readers, we analyzed the injury of AITFL and PITFL separately. Overall, MRI showed no discriminatory power for DTSI (Table 2) (*P* < 0.05) with syndesmotic injury (AUC values of AITFL injury were 0.517 for reader 1, and 0.529 for reader 2; AUC values of PITFL injury were 0.565 for reader 1, and 0.565 for reader 2). A multi variable analysis model with fixed and independent factors confirmed that AITFL (*P* > 0.05) and PITFL (*P* > 0.05) were not statistically significant for the diagnosis of DTSI, while DTJE (*P* < 0.05) was statistically significant for the diagnosis of DTSI (Table 3).

**Table 2:** Mean AUC values for accuracy in characterization of syndesmosis and DTJE for each reader and each method

	Routine MRI				Height		Projected area of the “equal-point” method		Projected area of the “incremental-value” method	
		AUC	95% CI		AUC	95% CI	AUC	95% CI	AUC	95% CI
Reader 1	AITFL	0.517	0.430 ~ 0.616	Group A	0.753	0.682 ~ 0.824	0.778	0.706 ~ 0.850	0.761	0.690 ~ 0.833
	PITFL	0.565	0.480 ~ 0.651	Group B	0.805	0.737 ~ 0.874	0.803	0.732 ~ 0.873	0.804	0.734 ~ 0.874
Reader 2	AITFL	0.529	0.441 ~ 0.616	Group A	0.762	0.692 ~ 0.832	0.783	0.712 ~ 0.854	0.763	0.692 ~ 0.834
	PITFL	0.565	0.479 ~ 0.653	Group B	0.811	0.743 ~ 0.874	0.817	0.747 ~ 0.887	0.805	0.735 ~ 0.875

*P* < 0.05 for all comparisons of AUC values between image sets and the value with 3 digits after the decimal point. *AUC*, area under the curve; *DTJE*, distal tibiofibular joint effusion; *AITFL*, anterior inferior tibiofibular ligament; *PITFL*, posterior inferior tibiofibular ligament; *CI*, confidence interval

**Table 3:** A multi-variate analysis model with fixed and independent factors was used to compare AITFL/PITFL/DTJE to a dichotomous endpoint (presence or absence of DTSI by arthroscopy)

Item		*P*	Item		*P*
Reader 1	AITFL	0.724	Reader 2	AITFL	0.846
	PITFL	0.096		PITFL	0.369
	DTJE	0.000		DTJE	0.000

*AITFL*, anterior inferior tibiofibular ligament; *PITFL*, posterior inferior tibiofibular ligament; *DTJE*, distal tibiofibular joint effusion; *DTSI*, distal tibiofibular syndesmosis instability. *P < 0.05

### Diagnostic efficiency of qualitative index of DTJE in MRI

The *κ* value representing the degree of interobserver agreement for the two readers with the qualitative index of DTJE was 0.845 (almost perfect agreement). We used 2 × 2 contingency table to judge the diagnostic efficiency of qualitative index of joint effusion. There were 133 patients with DTJE by reader 1 and 144 patients with DTJE by reader 2 in 212 cases (62 patients with DTSI). The result showed that sensitivity was 0.89, specificity was 0.48, Youden index was 0.37 with reader 1 and that sensitivity was 0.90, specificity was 0.41, and Youden index was 0.31 for reader 2 (Table 4).

**Table 4:** The 2 × 2 contingency table on diagnostic efficiency of qualitative index of DTJE

Item			Arthroscopic diagnosis		Sensitivity (%) (95% CI)	Specificity (%) (95% CI)	Youden index
			+	-			
Reader 1	DTJE	+	55	78	0.89 (0.78 ~ 0.95)	0.48 (0.40 ~ 0.56)	0.37
		-	7	72			
Reader 2	DTJE	+	56	88	0.90 (0.79 ~ 0.96)	0.41 (0.33 ~ 0.50)	0.31
		-	6	62			

*DTJE*, distal tibiofibular joint effusion; *CI*, confidence interval. *P < 0.05

### Diagnostic accuracy of the quantitative index of DTJE for DTSI

We employed ROC curve to rate the diagnostic accuracy of DTJE for DTSI. According to the classification criteria, both readers found that 16 patients had non-communication and reader 1 thought 117 patients belonged to the communication group but reader 2 assigned 128 patients to the communication group. Due to the small number of subjects in non-communication group, we divided 212 cases into a group A and a group B for statistical analysis. Group A consisted of communication group + non-communication group (16) + patients without DTJE, and group B included communication group + patients without DTJE.

**Fig. 6:**
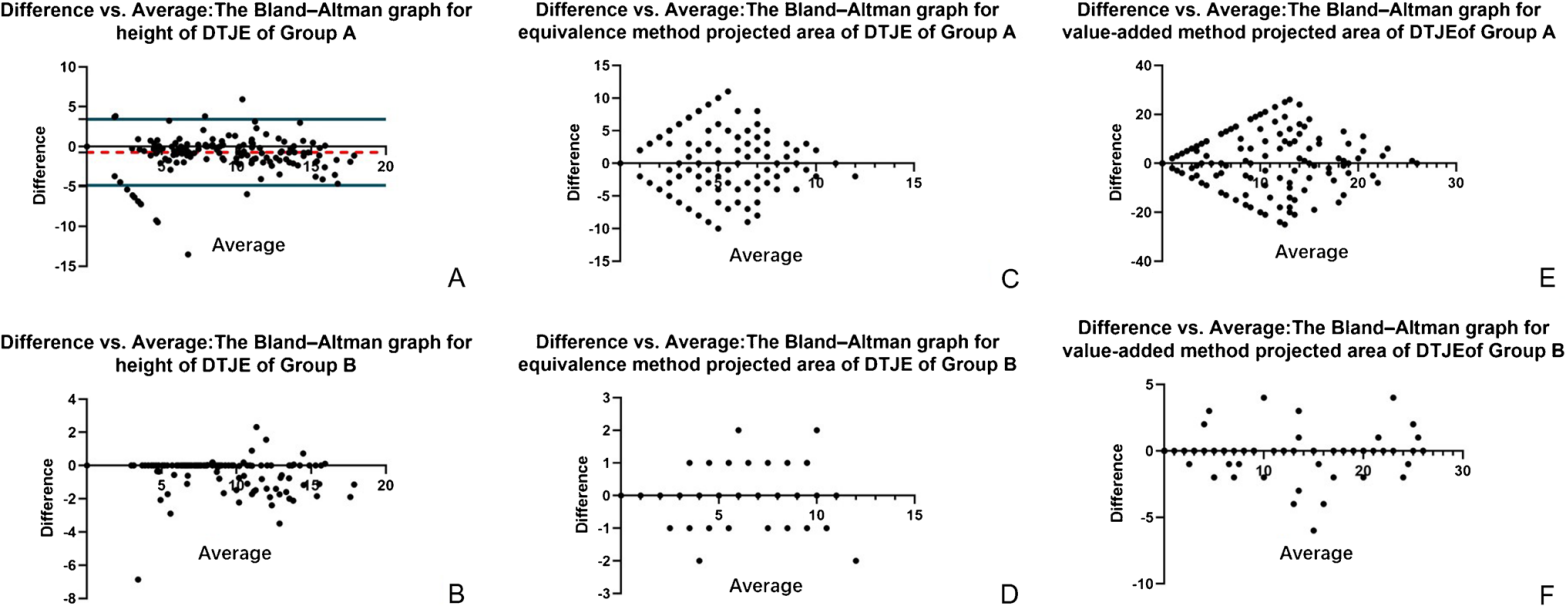
The Bland–Altman graph. **A** Height of DTJE for group A. **B** Height of DTJE for group B. **C** Equivalence method projected area of DTJE for group A. **D** Equivalence method projected area of DTJE for group B. **E** Value-added method projected area of DTJE for group A. **F** Value-added method projected area of DTJE for group B. DTJE, distal tibiofibular joint effusion

The Bland–Altman graph for height of DTJE revealed a bias of 0.72 mm and limits of agreement of [− 4.15; 4.15] for group A (Fig. 6A) and a bias of 0.27 mm and limits of agreement of [− 1.62; 1.62] for group B (Fig. 6B). The Bland–Altman graph for equivalence method projected area of DTJE revealed a bias of 0.05 and limits of agreement of [− 8.71; 8.71] for group A (Fig. 6C) and a bias of 0.01 and limits of agreement of [− 0.84; 0.84] for group B (Fig. 6D). The Bland–Altman graph for value-added method projected area of DTJE revealed a bias of 0.11 and limits of agreement of [− 20.08; 20.08] for group A (Fig. 6E) and a bias of 0.05 and limits of agreement of [− 1.90; 1.91] for group B (Fig. 6F).

**Fig. 7:**
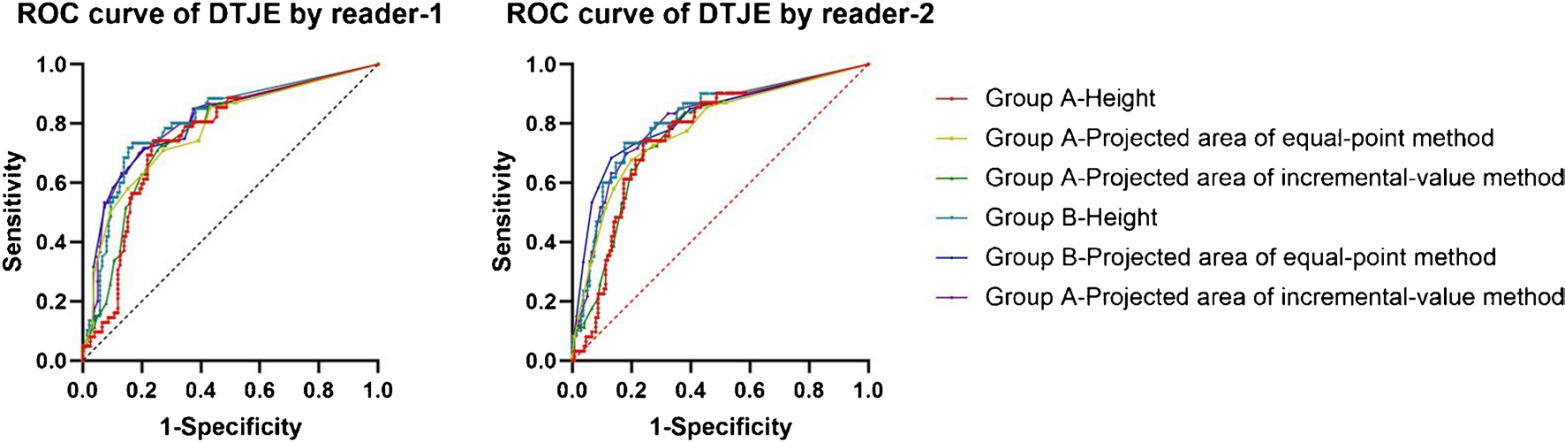
ROC curve of DTJE for different groups, indexes, and readers. The AUC values of the height/projected area of equal-point method/projected area of incremental-value method of DTJE were 0.753/0.778/0.761 for reader 1, 0.762/0.783/0.763 for reader 2 in group A and 0.805/0.803/0.804 for reader 1, 0.811/0.817/0.805 for reader 2 in group B. DTJE, distal tibiofibular joint effusion

There was significant difference between the two groups of the height/projected area of equal-point method/projected area of incremental-value method (Table 5). The AUC values of the height/ projected area of equal-point method/projected area of incremental-value method of DTJE were 0.753/0.778/0.761 for reader 1, 0.762/0.783/0.763 for reader 2 in group A and 0.805/0.803/0.804 for reader 1, 0.811/0.817/0.805 for reader 2 in group B (Table 2 and Fig. 7). The overall diagnostic accuracy for DTSI in group B was higher than in group A.

**Table 5:** Different measurement methods of DTJE on each reader

	Item	Height		Projected area of the equal-point m ethod		Projected area of the incremental-value method	
		Group A	Group B	Group A	Group B	Group A	Group B
Reader 1	Patients with DTSI	9.25 ± 4.50	9.15 ± 4.53	6.27 ± 3.36	6.32 ± 3.39	12.92 ± 7.83	12.95 ± 7.95
	Patients without DTSI	4.38 ± 5.03	3.51 ± 4.35	2.69 ± 3.11	2.29 ± 2.95	5.38 ± 7.23	4.16 ± 6.19
	*t*	6.594	8.260	7.453	8.402	6.741	8.375
	*P*	< 0.001	< 0.001	< 0.001	< 0.001	< 0.001	< 0.001
Reader 2	Patients with DTSI	10.11 ± 4.60	10.02 ± 4.65	6.45 ± 3.42	6.50 ± 3.45	13.13 ± 7.78	13.05 ± 7.89
	Patients without DTSI	5.13 ± 5.35	4.18 ± 4.50	2.69 ± 310	2.23 ± 2.81	5.45 ± 7.30	4.18 ± 6.18
	*t*	6.411	8.293	7.801	9.134	6.832	8.476
	*P*	< 0.001	< 0.001	< 0.001	< 0.001	< 0.001	< 0.001

Group A consisted of communication group + non-communication group (16) + patients without DTJE. Group B consisted of communication group + patients without DTJE. *DTJE*, distal tibiofibular joint effusion; *DTSI*, distal tibiofibular syndesmosis instability. *P < 0.05

## Discussion

In clinical practice, we found that the signal range of DTJE shown on MRI was significantly higher in patients who were arthroscopically diagnosed as having DTSI than in patients without DTSI. Therefore, we suspected that the amount of DTJE might be a more accurate index for the diagnosis of DTSI. Putting the AITFL, PITFL, and DTJE into a multi variable analysis model with fixed and independent factors, we found that ligament injury has no clinical significance in the diagnosis of DTSI but DTJE may be a good indicator for diagnosing diseases.

In MRI, we measured and assessed the effusion amount by using a pre-set method. Our results showed that the DTJE height and projected area in patients with DTSI were significantly higher than in those without DTSI, the finding being consistent with those of previous studies [17]. At the same time, as a continuous variable, we used ROC to quantitatively assess the diagnostic utility of DTJE. It was found that the AUC was higher in group B than in group A, as revealed by multiple methods. Our study suggested that the non-communication could not be a characteristic sign of DTSI. In fact, in this case, the distal tibiofibular syndesmosis was not damaged or the scar healing after injury was functionally sufficient, thereby stopping the tissue fluid of ankle joint from further invading the distal tibiofibular joint. At this time, the appearance of DTJE was more likely to stem from interosseous ligament injury caused by ankle sprain.

Previous anatomical studies found that there is a recess in the distal tibiofibular joint when DTSI occurs, so DTJE was more likely to take on an umbrella-like or mushroom-like shape on the sagittal plane [3]. However, due to the anatomic irregularity of the distal tibiofibular joint and the technological limitations, it is impossible to reconstruct the 3D image of DTJE through MRI. We approximately replaced the volume with effusion height and the projected area, and found that both of them could function as a good indicator for the diagnosis of DTSI. We recommended the height measurement be used for the clinical diagnosis of DTSI, which was simpler and more accurate, and the ROC results exhibited that the AUC (0.805/0.811) was the largest when the cut-off value of the effusion height was 8.00 mm/7.99 mm and the Youden index was the best. Although the sensitivity (0.73/0.73) was lower than that with only the presence of DTJE being taken into account (0.89/0.90), the specificity (from 0.48/0.41 to 0.83/0.82) was significantly improved, which suggests that its utility in disease screening in clinical practice was also greater. MRI was the most reliable tool for diagnosing injuries of soft tissues, such as ligaments and tendons, but it could not dynamically evaluate the state of motion. On the basis of our findings, we believe that type I and type II distal tibiofibular syndesmosis injuries could be distinguished by measuring the height of DTJE on coronal MRI. To be more specific, the effusion height > 8 mm strongly indicates DTSI. Moreover, the measurement can be accomplished on ordinary MRI, which is less costly and more practical than enhanced MRI [18] or high magnetic field MRI [19], and can minimize the possibility of misdiagnosis.

As a special type of ankle injury, MRI diagnosis of DTSI poses a special challenge for researchers. Clinically, sports medicine doctors diagnose the injury of the distal tibiofibular syndesmosis by assessing the ligament injury. Our study showed that the strategy did not work well for the diagnosis of DTSI (AUC < 0.6) if the diagnosis was based on AITFL or PITFL injury alone. Randell et al. found that MRI yielded a positive result for syndesmosis injury in chronic injuries after 12 weeks in 83.3% of patients, against a 100% rate achieved within 6 weeks, and that the result might be progressively less reliable over time [6]. In our study, virtually, all patients had a medical history of more than 3 months, so we believe that the diagnosis of DTSI solely based on ligament injury is not desirable in clinical practice.

On the basis of prior studies and clinical observations, we believe that the abnormal effusion in the distal tibiofibular joint might be related to impaired ligament function following ankle sprains and the entry of ankle synovial fluid into the distal tibiofibular joint under pressure. Therefore, DTJE was more a special manifestation of DTSI. Calder J et al. believed that the measurement of height of fluid above the ankle within the interosseous membrane cannot differentiate severe ankle sprains from high ankle sprains involving the syndesmosis [20]. Ryan et al. believed that DTSI diagnosis could be established as long as DTJE was present [7]. After retrospective analysis of 212 cases, in this study, we found that the sensitivity of DTJE, as a diagnostic indicator, was 0.89/0.90, but its specificity was 0.48/0.41 and Youden index of DTJE was 0.37/0.31. The results showed that DTJE did not exclude those who were actually not ill. Therefore, we believe that, clinically, the presence of DTJE alone is not a good indicator for the diagnosis of DTSI.

In the study, we divided DTJE into a communication group and a non-communication group based on MRI results. Since the MRI was a tomography and the slice thickness of our conventional MRI was 3 mm, the scanning was, inevitably, not sufficiently exhaustive. It is possible that MRI cannot scan some layers of effusion with communication between distal tibiofibular joint and ankle joint. Increasing the amount of data or appropriately reducing the scan slice thickness could decrease the systematic error and improve the diagnostic accuracy. As mentioned above, the irregularity of the distal tibiofibular joint and the particularity of the MRI image rendered it impossible for us to quantify the damage by reconstructing the DTJE volume. We used the height of DTJE and the projected area, instead of the 3-D spacial volume. Data loss cannot be avoided in the process of data conversion.

We found that the age, weight, and BMI of patients with DTSI were significantly lower than those of patients without DTSI, and females were more likely to get DTSI, which was essentially consistent with our clinical observation. The results might be ascribed to the fact that patients with DTSI prefer sports activities, and female ligaments are inherently more flexible. Future biomechanical experiments may provide explanations to this fascinating phenomenon.

## Conclusions

Our research transforms a complex MRI multi-dimensional spatial data into a simple measurement process, and puts forward that the 8 mm of DTJE height can be used as the diagnostic cut-off value of DTSI through the statistical analysis of a large number of MRI data. It can greatly reduce the diagnostic difficulty of DTSI and can be used as a new diagnostic method in clinical practice.

## Data Availability

All data are available upon reasonable request from the corresponding author.
